# Tracing Antibody Repertoire Evolution by Systems Phylogeny

**DOI:** 10.3389/fimmu.2018.02149

**Published:** 2018-10-02

**Authors:** Alexander Dimitri Yermanos, Andreas Kevin Dounas, Tanja Stadler, Annette Oxenius, Sai T. Reddy

**Affiliations:** ^1^Department of Biosystems Science and Engineering, ETH Zurich, Basel, Switzerland; ^2^Department of Biology, Institute of Microbiology, ETH Zurich, Zurich, Switzerland; ^3^Department of Chemistry and Applied Biosciences, ETH Zurich, Zurich, Switzerland

**Keywords:** systems immunology, phylogenetics, antibody lineage, B cell evolution, Ig-Seq

## Abstract

Antibody evolution studies have been traditionally limited to either tracing a single clonal lineage (B cells derived from a single V-(D)-J recombination) over time or examining bulk functionality changes (e.g., tracing serum polyclonal antibody proteins). Studying a single B cell disregards the majority of the humoral immune response, whereas bulk functional studies lack the necessary resolution to analyze the co-existing clonal diversity. Recent advances in high-throughput sequencing (HTS) technologies and bioinformatics have made it possible to examine multiple co-evolving antibody monoclonal lineages within the context of a single repertoire. A plethora of accompanying methods and tools have been introduced in hopes of better understanding how pathogen presence dictates the global evolution of the antibody repertoire. Here, we provide a comprehensive summary of the tremendous progress of this newly emerging field of systems phylogeny of antibody responses. We present an overview encompassing the historical developments of repertoire phylogenetics, state-of-the-art tools, and an outlook on the future directions of this fast-advancing and promising field.

## Introduction

B cells are the foundation of humoral immunity and are defined by their characteristic B cell receptors (BCR, or secreted version: antibodies), which bind foreign pathogens and initiate effector functions, such as pathogen opsonization, neutralization, complement activation, and cellular cytotoxic and phagocytosis signaling (1). Antibodies are composed of two identical heavy chains and two identical light chains, where each chain consists of a variable region and a constant region. The variable regions dictate antigen-binding specificity (2), whereas the constant regions enable interactions with other molecular and cellular components of the immune system (1). Initial variable region diversity is encoded in the organism's genome through the presence of multiple V-, D- (heavy chain only), and J-gene segments, which pseudo-randomly recombine in both the heavy and light chain loci (3, 4). During somatic recombination, the variable regions can undergo further diversification due to deletions or insertions at the V-D and J-D junctions, rendering a potential theoretical amino acid diversity in humans and mice of >10^13^ (5–7). The region encompassing the last few nucleotides of the V-gene segment, the entire D-gene segment (in the case of heavy chain rearrangement), and the start of the J-gene segment is known as the complementary determining region 3 (CDR3), and has been shown to largely dictate antigen specificity (2).

Selective pressures are present during early B cell development to ensure binding specificity is not directed toward self-antigens through interactions with stromal cells in the bone marrow. This is done via deletion or induction of anergy in B cells expressing BCRs exhibiting self-reactivity. B cells surviving this selection emigrate from the bone marrow and enter the circulating population of mature B cells. These newly produced B cells circulate between blood and secondary lymphoid organs until encountering their respective antigen. The BCRs which bind their respective target can subsequently engulf the foreign antigen via receptor-mediated endocytosis and display these pathogen-derived peptides on the cell surface using major histocompatibility class (MHC)-II proteins (8, 9). This prepares the B cell for further differentiation via binding of CD4+ T cells, which interact specifically with the foreign peptides displayed on the B cell's MHC-II molecules. Both the strength and duration of this interaction between B and T cells have been implicated in dictating the fate of the B cell (10). Longer conjugate interactions may preferentially lead to a germinal center (GC) reaction, where affinity maturation and class switching occur (11, 12).

**Figure 1:**
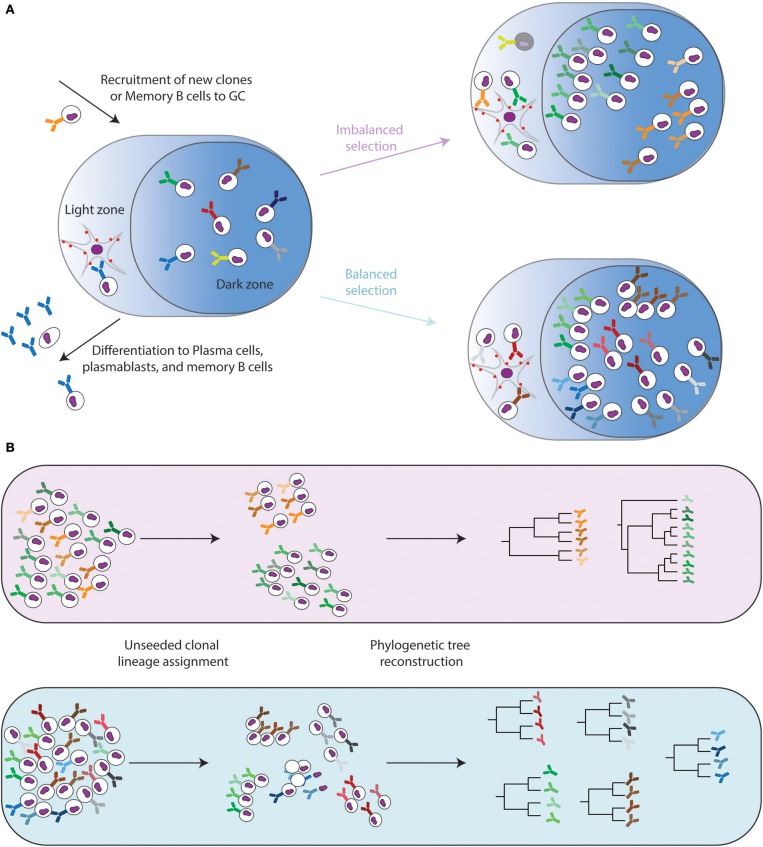
Evolutionary dynamics of the Germinal center reaction. **(A)** Naïve and memory B cells are recruited into germinal center reactions where they undergo subsequent rounds of somatic hypermutation in the dark zone and selection via follicular dendritic cells in the light zone. This leads to successive rounds of division and mutations (shown by colored antibody receptors) or apoptosis (shown by gray cells). Different selection pressures can lead to either balanced selection, in which multiple independent clones expand and undergo SHM, or imbalanced selection where a few clones dominate the GC reaction and undergo many rounds of SHM. **(B)** Ig-Seq can capture the sequence diversity within populations of B cells. Systems phylogeny aims to assign the recovered sequences into clonal families, followed by the inference of evolutionary histories. The resulting phylogenetic trees can then be compared both within one host and between hosts.

GCs are structurally divided into a dark zone, where B cells rapidly proliferate while mutations are selectively introduced into the antibody locus, initially via the enzyme activation-induced cytidine deaminase (AID) and the upregulation of the error-prone DNA polymerase eta (13–15), a process referred to as somatic hypermutation (SHM) (16). A number of reviews exist describing the complex biochemistry underlying SHM and are available for further reading (17, 18). The light zone in GCs is where T follicular helper (TFH) cells mediate the selection of B cell clones with higher antigen affinity and their differentiation into plasma cells (Figure 1A) (12, 19, 20). B cell clones incurring SHM that increase the strength of the antibody-antigen binding interaction will subsequently receive more survival signals, such as ICOS, CD40, and interleukin-21 (IL-21) (11, 21, 22).

It has been shown that antibodies surviving the selective pressures faced during affinity maturation are capable of producing high affinity antibodies with binding disassociation constants (Kds) hundreds to thousands of times higher than their germline progenitor (23). Furthermore, recent work in mouse models of chronic viral infection have revealed that the continued presence of TFH cells is crucial for the development of neutralizing antibodies (24). While it is intuitive that affinity maturation holds an essential role to improve the specificity and affinity of B cells against complex antigens (such as pathogens and their proteins), a recent study has questioned this, as it was proposed that there is a continuous recruitment of naïve or memory B cells equipped with high affinity BCRs into an ongoing humoral immune response (25). This suggests that SHM might play a prominent role in broadening the antibody response with respect to its ability to recognize antigenic variants (26, 27). Despite these recent findings, the exact nature regarding whether and how affinity maturation instructs antibody evolution remains at the forefront of contemporary antibody repertoire research. What recent studies have made abundantly clear, however, is that B cells with unique V-(D)-J rearrangements exist contemporarily, both within an organism and even within a single germinal center (Figure 1B) (27, 28). The utilization of new experimental techniques (e.g., multiphoton microscopy, confetti mice, and bone marrow chimeras) in concert with sequencing technologies have provided an unprecedented insight into how biological factors such as BCR affinity or clonal diversity can influence the evolutionary landscape.

Over the past decade, many fields of research have leveraged the increased resolution and decreased cost of high throughput sequencing (HTS) to better understand genomic diversity and evolution. Similarly, the field of immunology has employed HTS to investigate the genetic diversity of antibody variable regions, also referred to as immunoglobulin sequencing or Ig-Seq. This application has been instrumental in providing a quantitative description and profile of antibody repertoires (29–31). Ig-Seq experiments capture the diversity found in the variable regions of co-existing antibodies, enabling the reconstruction of multiple antibody lineages within a single host over time (32–34). Given the immense wealth of sequencing data arising from Ig-Seq, phylogenetic inference is a well-suited methodology to better understand clonal selection and expansion mechanisms that drive B cell evolution.

The standard evolutionary analysis of a B cell involves the reconstruction of a phylogenetic tree, in which the temporal relationships between recovered antibody sequences are modeled. The phylogenetic tree is often referred to as a clonal lineage, whereas a “phylogenetic lineage” represents a branch in the tree. In the case of antibody repertoire phylogenetics, each phylogenetic tree represents a clonal lineage descending from an independent V-(D)-J recombination event. From a single Ig-Seq experiment, a multitude of phylogenetic trees can be inferred, demanding a novel analysis pipeline not typically required in conventional phylogenetic studies examining species or viral evolution. The sequencing reads covering the full V-(D)-J region (~350–400 base pairs) are represented as nodes in the tree, while the edges indicate the relationship between the tips, and the edge lengths represent the time between branching events. These representations provide valuable information regarding the evolutionary history of a given antibody or B cell clone and can be employed to understand the selective pressures experienced during affinity maturation.

Studying how antibodies evolve in the context of pathogen neutralization has the potential to both answer basic biological questions pertaining to clonal selection and to aid in the development of precision vaccines or discovery of therapeutic monoclonal antibodies. Extensive research efforts have already been dedicated to better comprehend a subset of antibodies capable of neutralizing the infectious potential of multiple strains of HIV-1 (broadly neutralizing antibodies, bNAbs) (35–38). A prominent example involves the VRC01 bNAb lineage, originally identified from B cells of an HIV-1 patient, which has been shown to neutralize 90% of HIV-1 strains after undergoing extensive SHM (39). Using traditional phylogenetic methods, the evolutionary steps preceding virus-neutralizing capability were inferred, enabling the inference of both ancestral and intermediate sequences (38, 39). Further work has attempted to design vaccine immunogens that target these intermediate progenitor sequences in hopes of directing the subsequent evolution of antibodies toward the broadly neutralizing phenotype (40, 41). Additionally, how affinity, avidity, and the initial concentration of these progenitor BCRs influence the subsequent GC reactions and incurred mutations was recently described, providing further insight about the appearance and propagation of bNAbs (42).

While the various HIV-1 bNAbs have ignited hopes of utilizing phylogenetics to design vaccines for rapidly mutating viruses, most research employing antibody phylogenetics has been confined to single clonal lineages (35–37, 43, 44). Despite the emphasis on single antibody lineages, the majority of the sequencing data used to describe these neutralizing antibodies has been recovered via Ig-Seq experiments. Thus, while individual trees describing the evolution of HIV-1-neutralizing antibodies have been well characterized, several unanswered questions remain regarding how to partition the sequencing reads into the individual V-(D)-J recombination trees, and how this antibody “forest” of distinct phylogenetic trees evolves as a system.

The unique opportunity to apply sequencing technologies to the study of B cells has led to the development of several tools and practices specifically tailored to the investigation of antibody evolution (45–47). It is foreseeable that this trend will only continue to increase as Ig-Seq experiments become increasingly commonplace in immunological research given the applications both to antibody therapeutics and rational vaccine design (48). Despite the lack of standardization, many studies have already incorporated phylogenetic analyses in concert with Ig-Seq (34, 38, 49). These studies have employed various tools, inference methods, and heuristics. We provide here a comprehensive review tailored specifically to antibody repertoire phylogeny. We outline both contemporary practices and software, in addition to the problems currently faced by this promising field.

## Clonal lineage assignment

As opposed to traditional phylogenetic studies, the somatic diversification mechanisms inherent to B cell development present an additional pre-processing step even before the selection of a tree-inference method. V-(D)-J recombination creates an immense starting pool of roots, each of which has the potential to encounter its cognate antigen and subsequently undergo clonal expansion and evolution (polyclonal response). Therefore, at any given point in a single individual host, multiple co-evolving lineages will be present. Phylogenetic analyses involving pathogens traditionally assume that all recovered sequences are related to a single common ancestor. Thus, correctly assigning a given B cell clone to a particular clonal lineage presents a challenge not found in other phylogenetic analyses. Upon successfully sequencing the B cell populations of interest, the recovered reads need to be first assigned to a given phylogenetic tree, representing a group of clones expanded from a single V-(D)-J recombination event (Figure 1B). A given Ig-Seq experiment can produce millions of sequencing reads per sample (4, 29, 50), rendering it difficult to disentangle the simultaneous, independently co-evolving lineages. Several strategies and tools have been recently developed in response to this problem and are outlined below.

A common starting approach is to initially cluster sequences by their germline genes, and subsequently infer an individual tree for each cluster. Based on the number of possible combinations of V-, D-, and J-genes, this implies that thousands of phylogenetic trees could be inferred within a single individual. In practice, not all germline genes and combinations thereof are used at the same frequency, which dramatically reduces the number of actual trees produced within one host (4, 51). Additionally, low alignment accuracy of the D-gene segment has led many studies to only consider the V- and J-gene segments during clustering. The number of trees within a single individual can be further reduced by setting a threshold for a number of sequences per tree. Unfortunately, the value to define the threshold is less clear and often depends on the context of biological questions. For example, there exist studies which have set thresholds of 10 sequences per tree when tracing B cells across various compartments (e.g., B cells trafficking to the central nervous system) (52), whereas other studies that depict differentiated memory B cells within a tree have omitted a threshold altogether (49). In addition to lower limits set on the number of sequences required per tree, upper limits can also be set depending on the computational demands of the selected phylogenetic method. Multiple HIV studies, for example, have restricted each lineage tree to a maximum of 200 randomly sampled sequences for the root of interest (36, 43).

The challenge of assigning reads to a clonal lineage can be addressed by taking advantage of the nature of SHM to preferentially introduce nucleotide substitutions during GC reactions (53). This implies that insertions and deletions are mainly introduced via V-(D)-J recombination. Therefore, information regarding insertions and deletions can be utilized to restrict sequences with identical clonal (CDR3) lengths to a given tree. This dramatically increases the number of trees per individual, while decreasing the number of sequences assigned to a given clonal lineage. Under the assumption that clonal lineages evolve independently, phylogenetic trees from a particular individual can be computed in parallel. Thus, this heuristic approach can dramatically reduce the necessary computation time while incorporating relevant biological insight regarding a constant CDR3 length throughout the affinity maturation process.

Commonly used tools capable of aligning Ig-Seq data are MiXCR, IMGT, IgBlast, SONAR, IGoR, iHMMunealign, and Partis (54–60), which work by assigning germline genes to sequencing reads and additional annotation [Framework regions (FRs) and CDRs] (Table 1). In some cases, such as with MiXCR, Partis, and IgBlast, a user is able to include a custom reference germline database (particularly useful in cases where germline genes of a given species have not yet been fully annotated) (54, 56, 57); this can be used in concert with software capable of predicting germline alleles from Ig-Seq data. While Partis has this capability built in (61), other standalone software includes IgDiscover and TigGER (62, 63). Additionally, one can extract germline information from whole genome shotgun sequencing, as performed by VGeneRepertoire (64). One of the major drawbacks of the previously mentioned lineage assignment is the large reliance on an initial alignment of recovered reads to the germline. Furthermore, any rare insertions or deletions introduced during SHM will be excluded due to restricting trees to an identical clonal (CDR3) length.

**Table 1:** Comparison of tools and methods used for clonal lineage assignment and phylogenetic inference.

	**Attributes (+)**	**Notes (–)**
**Clonal LINEAGE ASSIGNMENT**		
Alignment based (Mixcr, IMGT, IgBlast, IGoR, IHMMunealign)	Potentially fast run time (depends on the tool) Can often supply own germline genes	Often arbitrary thresholds for clonal relatedness (e.g., 80% CDR3 similarity)
Partis (https://github.com/psathyrella/partis)	Human, mouse and macaque germline built in Germline inference possible Docker image available Good documentation	Large datasets may require subsampling due to computational demands
Clonify (https://github.com/briney/clonify-python)	Antibody specific edit distance Explicit incorporation of shared mutational histories	Limited to unseeded alignment
SONAR (https://github.com/scharch/SONAR)	Multiple seeded lineage assignment algorithms Easy export to other phylogenetic software Docker image available	Limited to Human germlines
**PHYLOGENETIC METHOD**		
Distance based (ape, ClustalOmega, EBI, phangorn, FastML)	Computational speed Multiple distance metrics possible	Difficult to calculate distances for sequences with large divergence and alignment gaps Less sophisticated than probabilistic methods
Maximum parsimony (PHYLIP, Rphylip, GCTree, phangorn, IgTree)	Intuitive algorithm Clonal frequency incorporation (GCTree) Polytomies and internal nodes (IgTree)	Ignores antibody specific properties (hotspots, transversions, transitions) Long-branch attraction problem
Maximum likelihood (FastML, MEGA, IQ-TREE, dnaml, IgPhyML)	Complex substitution models Hotspot specific codon models (IgPhyML)	Computationally demanding Sensitive to model misspecification
Bayesian (BEAST, Mr. Bayes, ImmuniTree)	Complex substitution models Can produce rooted trees without explicit outgroup Possible to incorporate biological knowledge with priors Mutation rate returned in calendar time (BEAST)	Sensitive to model misspecification Highest computational demands due to Markov chain Monte Carlo algorithm

Several methods have been developed to circumvent problems arising during alignment-based lineage assignment. These methods include both seeded and unseeded lineage assignment. Seeded lineage assignment aims to extract all clonally-related transcripts to an input antibody sequence. Conversely, unseeded lineage assignment attempts to decompose the entirety of input sequences into their constitutive clonal families. Three prominent tools specifically tailored to clonal lineage determination are Partis, Clonify, and SONAR (57, 58, 65). Partis models B cell evolution with a likelihood function that avoids the need to strictly define rooting assumptions, such as an arbitrarily defined percentage of CDR3 sequence homology (57). This tool can perform both unseeded and seeded lineage assignment, with input sizes reaching hundreds of thousands and millions of sequences, respectively. Another tool, Clonify, uses hierarchical clustering based on an antibody specific edit distance to determine clonal lineage inclusion (65). One benefit of this proposed algorithm relative to the aforementioned alignment tools is that neither CDR3 lengths nor germline alignments explicitly define a clonal lineage. Instead, CDR3 similarity, germline alignment scores, and information regarding shared mutational histories are included in the clonal assignment. Finally, SONAR first aligns reads to germlines provided by IMGT and can subsequently perform either seeded or unseeded lineage assignment (58). Their unseeded alignment relies upon first separating transcripts into groups based on V- and J- genes, with subsequent clustering based on CDR3 sequence similarity. Multiple algorithms for seeded lineage assignment are available, in addition to functions which allow visualization of homology to germline genes and other known antibodies (58). While the subsequent phylogenetic tree inference is possible with SONAR, clonal lineages can also be easily exported to formats compatible with other commonly used tree inference software. Finally, both Partis and SONAR are available as Docker containers, which can dramatically simplify the installation process. While these methods are a promising step to improve the delineation of independent V-(D)-J recombination events from bulk sequencing data, further benchmarking studies are still required to illustrate how clonal lineage assignment algorithms influence the downstream evolutionary conclusions. Such studies, for example, could examine how the amount, topologies, and sizes of lineage trees from a single repertoire change based on preprocessing and lineage assignment pipelines.

## Structure of the B cell tree

**Figure 2:**
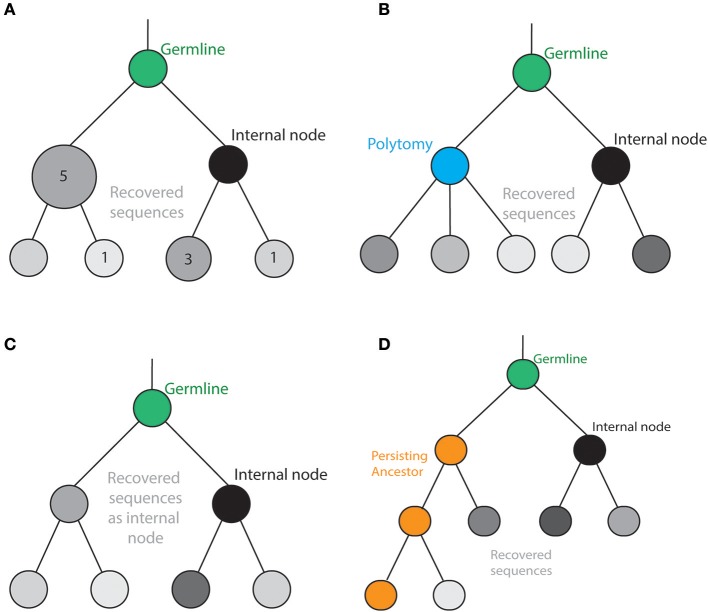
Tree topologies for B cells. **(A)** The inclusion of polytomies in the phylogenetic tree allows a B cell to produce more than two distinct offspring at a given internal node. **(B)** Experimentally recovered sequences can be inferred as either internal nodes or tips in the phylogenetic tree. **(C)** Persisting ancestral sequences can be sampled at multiple time points while also producing distinct offspring. **(D)** Clonal frequencies have often been illustrated by the size of the nodes. Therefore, information regarding clonal expansion can be incorporated into the resulting topologies.

Phylogenetic trees are commonly defined such that each node represents a recovered B cell sequence (or clone), whereas the branches represent the relationship between sequences. However, there exist several important differences between traditional phylogenetic trees and models specifically tailored to describe B cell evolution (Figure 2). One important characteristic of B cell maturation is clonal selection during expansion, which results in multiple B cells that have identical BCR sequences. Therefore, Ig-Seq can return identical reads corresponding to different B cells, adding a frequency attribute to each recovered sequence. The most common method currently employed by repertoire studies has been to remove replicate sequences, producing a phylogenetic tree entirely composed of unique sequences. However, this approach is inherently biased given the disregard for clonal expansion, a biological phenomenon seminal to B cell immunity. In particular, evolutionary rates are over-estimated as the periods without mutation during clonal expansion are disregarded.

Furthermore, it has been recently shown that the starting amount of antigen-specific memory (precursor) B cells (i.e., ancestral sequences) in a given lineage directly impacts the ability to engage in GC reactions and undergo further mutations (42). This stresses the importance of implementing phylogenetic methods that can incorporate clonal frequencies into the tree reconstruction calculation. To account for clonal expansion, antibody studies have displayed phylogenetic trees where the size of the node refers to the number of identical sequences (Figure 2A). While this leads to a visual representation of clonal abundance, this information does not contribute to the phylogenetic inference, thereby ignoring valuable information describing the evolutionary processes underlying clonal selection. Therefore, recent progress has been made to combine traditional phylogenetic inference methods with this clonal abundance data (66). In what are referred to as GCTrees, clonal abundance information was explicitly modeled into the phylogenetic inference process, leading to increased accuracy based on simulated trees (66). Furthermore, this reconstruction method allows for the inclusion of recovered sequences to serve as internal nodes (for the rationale, see section The Mutation Process Along the Tree) (66). This methodology highlights the progress toward integrating the biologically relevant information recovered from Ig-Seq experiments into the reconstruction of antibody phylogenies.

The traditional phylogenetic framework produces trees where the recovered sequences are positioned as leaves of the trees. However, there are several antibody evolution studies that have conceptualized the internal structure of the phylogenetic tree to better suit B cell evolution and selection. This involves the allowance of polytomies (more than two descendants from a single internal node) and intermediate sequences serving as internal nodes (Figures 2B,C). The underlying logic behind this dramatic shift from traditional evolutionary studies relies on the assumption that a given B cell clone can produce multiple distinct offspring (somatic variants), each of which may be separated by only a single mutation. Furthermore, this same ancestral B cell may persist long after giving rise to progenitor cells without incurring further mutations (Figure 2D). To account for both of these biological considerations, antibody-specific phylogenetic tools such as IgTree and ImmuniTree allow for both the presence of polytomies and the presence of recovered sequences as internal nodes in the resulting lineage tree. While these topological frameworks diverge from traditional phylogenetic analyses, they introduce a flexibility that allows for the incorporation of antibody-relevant information. However, it remains unknown how these adjustments to the phylogenetic model tree impact the biological conclusions such as tree shape and mutation rates. It would be interesting to investigate into how the tree structure of HIV neutralizing antibodies, for example, would change if polytomies were allowed in the phylogenetic reconstruction.

## The mutation process along the tree

The enzymatic nature of how AID induces mutations during affinity maturation dictates the evolutionary trajectories possible for a given B cell. AID introduces mutations by preferentially targeting the immunoglobulin locus via the deamination of deoxycytidine residues into deoxyuridines. This newly introduced deoxyuridine results in a mismatch pairing in the DNA and is subsequently corrected by either MMR or BER. The majority of mutations introduced after these nucleotide repair pathways are in the form of point mutations, although there are occasional deletions or insertions present (67, 68). These substitutions must not only maintain stability of the BCR, but also provide a functional antibody capable of surviving antigen selection imposed during GC reactions (Figure 1A). This selection has been implicated in improving binding affinity, broadening of antigen recognition and the development of specific effector functions such as pathogen neutralization (24, 39). Interestingly, the shift from pathogen binding to pathogen neutralizing is not always associated with a large increase in binding affinity, suggesting a more nuanced role of affinity maturation than solely promoting high affinity antibodies (69).

Given that mutations are introduced through enzyme-mediated mechanisms, it is somewhat intuitive that particular patterns in the genome would be preferentially targeted. Even before the advent of HTS, certain nucleotide motifs, termed “hotspots,” have been demonstrated to incur point mutations at greater than average frequency (70). One initial example supporting this neighbor-dependent model of SHM was the discovery of the RGYW motif (where W = A/T, *R* = A/G, Y = C/T), where the adjacent nucleotides influence the mutability of the central G nucleotide (70). Subsequent experiments uncovered additional motifs targeted by AID, albeit at low numbers due to limitations arising from low-throughput experimental settings (71–73). However, recent studies employing Ig-Seq have provided a thorough analysis of how neighboring nucleotides influence the probabilities of point mutations (74, 75). One prominent example compared synonymous and non-synonymous mutations across multiple Ig-Seq datasets to infer mutational probabilities for 5mers (nucleotide sequences with length 5), termed the “S5F” model (74). This substitution model contains inferred transition probabilities for the middle nucleotide of all possible 5mers, both verifying historical, low-throughput experimental data, and discovering novel motifs. In subsequent work, similar models were developed to describe the specific mutational properties of the 5mer motifs found in light chains arising from human and mouse data, providing a wealth of pertinent information to the mutational landscape of SHM (75). The refinement of distinct hotspot models for heavy and light chain evolution is crucial because the inference of heavy and light chain phylogenies can be performed separately, as performed in studies comparing the evolution of heavy and light chains in the context of HIV infection (38). However, when the pairing of heavy and light chains is known, the loci can be combined (concatenated to each other) and treated as a single evolving entity. This can increase the information used when inferring evolutionary parameters such as mutation rates and tree structure, given that both loci must share the same tree topology. Despite these findings describing the neighbor-dependent nature of AID, most modern phylogenetic methods rely on the assumption of site-independent substitution models, in which the neighboring nucleotides play no role in the evolutionary inference calculation. Thus, current studies analyzing B cell lineages typically do not account for this well-established biological phenomenon that may also have evolutionary ramifications.

One promising step to incorporate the properties of SHM hotspot motifs into the phylogenetic inference process has been demonstrated by the implementation of the HLP17 codon substitution model, which accounts for neighbor-dependent hotspot mutations, germline sequence knowledge, and irreversible evolution (76). This substitution model (available in the IgPhyML program) has been shown to perform better on Ig-Seq data than conventional phylogenetic substitution models because of the inclusion of phylogenetic inference parameters that describe the WRC hotspot (76). Specifically, it could be observed that the use of this codon model reduced bias in evolutionary parameters such as tree length (76), which has been previously shown to be difficult to estimate for multiple bNAb lineages with traditional substitution models (38). Their model allows for any motifs of length three nucleotides to be incorporated while still assuming that these hotspot motifs (i.e., codons) evolve independently to maintain computational feasibility (76). While all motifs cannot yet be explicitly accounted for simultaneously due to computational limitations, this work represents important progress toward incorporated motif-specific properties of SHM. One additional drawback remains that this substitution model is not yet available in many commonly used phylogenetic tools, potentially limiting its application.

## From sequences to trees

Multiple phylogenetic inference methods exist to construct the antibody lineages, each of which have their strengths and weaknesses (Table 1). A variety of these methods have been employed for the analysis of Ig-Seq data, including distance-based methods (44, 45, 77), maximum parsimony (36, 52, 78, 79), maximum likelihood (37, 43, 44, 80, 81), and Bayesian inference (38, 47, 82). Most methods initially require a multiple sequence alignment (MSA), which allows for lists of sequences with varying lengths to be compared in a site-dependent manner. Some common examples of MSA tools include ClustalOmega, Kalign, MUSCLE, and T-coffee (83–86). The output of the MSA file will usually be in fasta, nexus, or phylip format, which is easily integrated with the phylogenetic reconstruction methods described below.

### Distance-based methods

Distance-based methods involve first filling a matrix by an all-against-all calculation of a given metric comparing pairwise sequence similarity (87). The distances between sequences are often calculated using a substitution model. This allows for the incorporation of certain characteristics of sequence evolution, such as indicating different rates of evolution for transitions (purine <-> purine, pyrimidine <-> pyrimidine), and transversions (purine <-> pyrimidine), as well as taking into account the possibility of hidden mutations (such as backward mutations). A neighbor-joining algorithm is utilized to produce the tree, which involves successively joining two sequences together with newly created internal nodes (88, 89). One major advantage of this method is that tree inference is very fast. Therefore, this method can be especially useful for exploring large Ig-Seq data sets, particularly when there are many sequences in each lineage tree. A noteworthy example of this implementation was seen when examining the evolution of HIV-1 bNAbs, in which the neighbor-joining method was used exclusively for large datasets (45). There exist many tools that can produce neighbor-joining trees, either found online with ClustalOmega or EBI bioinformatics server, in addition to R packages such as phangorn or ape (84, 90, 91). One notable example of a distance metric that does not require a MSA is the Levenshtein distance. The Levenshtein distance describes the number of changes (mutations, insertions, or deletions) required to change one string into another, and has been used extensively in Ig-Seq experiments in the past (4, 92).

### Maximum parismony

Another non-parametric method of inferring antibody evolution involves the use of maximum parsimony, in which the output phylogeny is the tree that can explain the evolution with the least amount of mutations (93, 94). This method does not allow for the incorporation of parameters specific to antibody evolution, which can be a disadvantage when there is abundant background knowledge of the experimental system. Conversely, the lack of assumptions regarding the substitution process may prevent model misspecification and thereby erroneous conclusions. Maximum parsimony has been used in multiple studies pertaining to Ig-Seq data, with some notable examples, examining B cell migration to the cervical lymph node or the development of neutralizing antibodies against West Nile virus (4, 74). Several tools exist to create maximum parsimony trees, although the most common among them is PHYLIP (95). Additionally, R packages such as Rphylip and phangorn have both incorporated maximum parsimony, allowing one to work within the R framework (91, 96). Finally, as previously stated, the GCTree utilizes a modified maximum parsimony to allow for clonal frequencies to influence the phylogenetic inference (67).

One of the earliest methods specifically tailored to inferring antibody evolution, IgTree, utilized a customized parsimony metric to produce lineage trees (45). This tool additionally introduced the concept of inferred intermediate sequences, in which all direct ancestral sequences were restricted to the separation of a single mutation (46). For example, two “inferred” internal nodes would be created when two sequences differing by three nucleotides are in the same clonal family. Thus, even if an intermediate sequence was not explicitly sampled, there would be a corresponding internal node in the output phylogeny. IgTree has been used to characterize how B cells evolve under a variety of selective pressures, such as lymphomas, multiple sclerosis, and autoimmunity (33, 77, 97).

### Maximum likelihood

Another method applied to study antibody evolution is maximum likelihood, which relies on the optimization of a likelihood function. This parametric method incorporates a substitution model that can dictate parameters such as nucleotide/amino acid frequencies and allow for different substitution rates for transitions and transversions. Thus, maximum likelihood can utilize evolutionary models that may better describe antibody evolution than the neutral assumption that all nucleotides are the same. Some of these models include the HKY, GTR gamma, and JC69 (98–100), which allow for nucleotide specific behavior (e.g., *A* mutating to *C* can have a different rate as *C* mutating to *G*). It may not be immediately apparent which substitution model best fits the data at hand, whereby tools that include model selection capabilities may be useful. Notable programs utilized in the context of Ig-Seq data include FastML, MEGA, IQ-TREE, and Phylip's dnaml (33, 90, 94–96, 98, 101–103). As mentioned above, one notable limitation of these substitution models is that the transition probability of a given site is independent to the neighboring nucleotides. Thus, building upon models which incorporate information regarding hotspot mutability represents a cornerstone of contemporary systems phylogenetics research (76).

A multitude of studies have employed the maximum likelihood method to analyze Ig-Seq data, with many focusing on the evolution of HIV-neutralizing antibodies (35, 39, 43, 44, 80, 37, 104, 105). Despite most maximum likelihood programs producing a “traditional” phylogenetic tree, where recovered sequences cannot serve as intermediate nodes and polytomies are absent, the biological relevance of these maximum likelihood trees has been proven by the inference and production of intermediate and ancestral germline sequences which possessed virus-binding capabilities (36, 40).

### Bayesian inference

The final considered method of phylogenetic inference relies upon Bayesian statistics, which is thus capable of incorporating prior biological information (known as priors) into the inference process. This includes information regarding the evolution of the B cells, in particular the mutation rate, and the replication of the B cells generating the tree, in particular B cell duplication and death rates. The most commonly used tool is BEAST (106, 107), which has many learning resources and user-contributed modules that are available for download. This method involves the largest computational demands compared to other phylogenetic methods both in terms of memory and calculation time (87). This largely is due to the inference process, which utilizes a Markov chain Monte Carlo (MCMC) algorithm to explore parameter space. This provides a sample from the posterior probability distribution, i.e., the output consists of millions of trees, which are a sample of the probability distribution. One can summarize this distribution into a single tree, termed as the most credible clade (MCC) tree, allowing for an easier comparison between multiple trees.

One further advantage of BEAST is that one can easily specify the time at which sequences were sampled, and that the output consists of trees with branch lengths in calendar time units (rather than number of substitutions as in all methods above). This kinetic information restricts the position of the sequence in the tree, in addition to inferring mutation rates in calendar time units. Thus, Bayesian methods present a strong advantage when time-resolved Ig-Seq data is available. One major drawback is the limited number of sequences that can be included in each phylogenetic tree, as trees with more than ~500 antibody sequences often require substantial computation time (e.g., months on a server) and do not always converge to the posterior distribution. Furthermore, if many lineage trees are desired, running the MCMCs in parallel is essential given the slow computation time. BEAST has been used to infer mutation rates of neutralizing antibodies and subsequently compared to viral evolution (39). An interesting result from this analysis was that the heavy and light chains evolved at similar rates for this particular bNAb. Furthermore, it was shown that different neutralizing antibody lineages evolve at different rates, suggesting multiple mechanisms underlying the maturation of bNAbs.

An antibody-specific tool, ImmuniTree, has been developed that incorporates a Bayesian framework into the inference of lineage trees (48). ImmuniTree allows for recovered sequences to be placed at internal nodes, polytomies, and accounts for spurious diversity arising from sequencing errors. Furthermore, the trees produced by ImmuniTree can depict the percentage of mutations a given immunoglobulin sequence has, thereby incorporating information not included in most other inference methods. Practically, this tool has been used to reconstruct lineages of bNAbs and to infer ancestral intermediates of these antibodies (47, 82). The phylogenetic data was subsequently used to direct experiments which displayed that the neutralizing breadth of these intermediate antibodies was still present, despite a lesser extent of SHM (48).

### Rooting the phylogenetic tree

The presented phylogenetic methods (with the Bayesian methods as exceptions) return trees without a root, i.e., the tree does not consist of information regarding on which branch the clonal expansion process started. Thus, these unrooted trees need to be rooted, which is typically done using an outgroup (for example, when inferring an ape tree, one can use a-non-ape primate sequence as an outgroup for rooting). For B cell phylogenies, we have knowledge regarding the underlying V-(D)-J recombination, meaning that unmutated V-(D)-J germline sequence can be incorporated into the tree reconstruction process as the outgroup. One major assumption of this strategy is that there is sufficient confidence in the germline annotations. This assumption may increase the information present during the phylogenetic analysis for inbred model organisms, such as mice or zebrafish. However, when the exact genomic composition of the V-(D)-J germline segments is unknown (e.g., in humans, where there are slight allelic changes in the germline between individuals), this discrepancy could alter the inferred mutation rate.

BEAST produces rooted trees even without explicitly designating any germline sequences as the outgroup. This can be advantageous when an exact annotation of the germline genes is lacking. While it is possible in BEAST to explicitly specify the root of a tree, it is not immediately straightforward due to the nature of the software. In the case where no germline sequences are supplied as a root, there exists an additional tool in the program that allows for the user to infer the sequence at the root (in addition to sequences at internal nodes). Important to note, however, is that the accuracy of this method has not yet been explicitly validated for antibody evolution (i.e., compared unmutated ancestors inferred from BEAST to the known germline sequences). Further benchmarking on both simulated data and experimental data is required to better understand how rooting with the germline segments influences the subsequent biological conclusions, for example mutation rates and topology metrics.

### Simulations

Simulations of antibody evolution represents a powerful approach to validate and explore the consequences of various phylogenetic tools and heuristic strategies. Initial antibody repertoire simulation frameworks did not possess a temporal component (i.e., no explicit rate at which sequences change in regard to time), hence preventing the investigation of how traditional phylogenetic methods perform on Ig-Seq data (108). Recently, multiple tools have been developed to account for evolutionary properties specific to B cell evolution. Elements such as hotspot motifs, clonal abundances, and mutation rates can be defined to produce an output phylogenetic tree along with the accompanying mutated sequences. These sequences can then be fed as input into various phylogenetic inference methods to validate tree reconstruction accuracy. Tree accuracy is validated by comparing the inferred to the simulated tree, e.g., via the Robinson Foulds distance, clade accuracy, and treescape metrics (46, 109, 110). While simulations are commonly incorporated in Ig-Seq experiments, these are largely in-house and not always publically available. An important step to improve benchmarking tools and strategies for Ig-Seq experiments includes making these simulation platforms publicly available to increase their use.

## Downstream analysis

One of the difficulties of including phylogenetic trees into Ig-Seq studies is the extraction and interpretation of biologically relevant conclusions. An emerging trend has been to focus on a few select lineages and leave the majority of the repertoire unanalyzed. Thus, major questions regarding how the entirety of the antibody repertoire evolves remain unanswered. The hurdles of inferring potentially hundreds to thousands of phylogenetic trees per individual is daunting both due to the computational demands and the subsequent analysis. Furthermore, comparing trees within a single host, and to other organisms introduces a further layer of complexity.

One of the most immediate results of phylogenetic inference is the output of a phylogenetic tree. The topology of these trees provides a visualization of the evolutionary relationship between a set of antibodies, which can be both qualitatively understood and quantitatively compared. Qualitatively, an imbalanced tree (defined as the two daughter lineages of a node have very different numbers of descending nodes) can be interpreted in that a single progenitor clone continuously out-survives the other clones. Thus, tree imbalance may describe the breadth of underlying selection pressures. This selective pressure where a single clone outcompetes the remaining population has been seen in other infectious species, for example influenza between hosts or HIV within a host (111). Conversely, when selection occurs evenly throughout a lineage, many clones may simultaneously proliferate, which can be observed as a balanced structure of the tree (Figure 1). Balanced trees have e.g., been observed for HIV between hosts (111). While Ig-Seq papers have mentioned these topological characteristics, few have thoroughly analyzed these phylogenetic structures. There exist metrics arising from the evolutionary biology field capable of describing tree topology in a way that allows comparison of the lineage trees both from within a single host and across individuals. Metrics such as the Colless number, the Sackin index, and the average number of ladders characterize tree “imbalance” (112, 113). Mathematically, these metrics account for the number of descendant sequences in right and left sub-trees at all internal nodes, producing a single value for the entire tree. This value can then be directly compared to other clonal lineage trees, providing a framework for a systems analysis of lineages. This concept of analyzing tree shape and imbalance has been implemented in the comparison of vaccine-responsive lineages to persistent lineages (highly abundant lineages that did not change in response to vaccination) (114). Lineages that were unresponsive to vaccination showed a more balanced evolution, whereas the vaccine-enriched lineages often had a focus on multiple positively selected subclones (114).

While the metrics above have not often been applied to Ig-Seq experiments, other topological metrics have been used to quantify clonal selection. For example, clonal lineage trees were produced to better understand the diversification processes underlying a subset of B cells residing in the bone marrow of human patients suffering from light chain amyloidosis (115). The downstream analysis described structural properties of the phylogenetic trees, such as the number of sequences per tree, the length of the trunk (distance from root to first branching event), pass-through nodes (internal nodes with a single offspring), the distance to the nearest branching event (thus quantifying how mutations separate a sequence's direct ancestor), and tree branching (determined by the outgoing number of internal nodes). Similarly, another study found that during gastric lymphomas, B cell evolution results in trees with longer trunks and path lengths when compared to gastritis, correlating with a higher initial affinity and a higher selection threshold (34).

While these structural motifs and tree-imbalance metrics provide a natural analysis of phylogenetic trees in biological terms, there additionally exist less intuitive metrics yet to be applied to Ig-Seq data. Phylogenetic trees are essentially acyclic graphs (graphs = networks), suggesting that novel methods in graph theory may potentially find their use in Ig-Seq studies. One potential example of utilizing graph theory arises from examining the Laplacian spectra of the many trees within an individual. This approach was suggested recently to possess a multitude of parameters describing individual tree shape and branch length in the context of eigenvector distributions (116). However, few studies have leveraged such topological analyses of unlabeled antibody trees, thus, the extent to which meaningful biological conclusions can be drawn remains unseen.

In contrast to qualitative topological analysis, statistically derived parameters may be of further interest to provide a quantitative description of the evolutionary process of antibody lineages. Traditionally, repertoire studies have been interested in counting the number of mutations found at a given time point, however, leveraging phylogenetics, one can quantify how often a given lineage accumulates mutations in a time-resolved fashion. As previously stated, Bayesian phylogenetics has already been utilized to calculate the mutation rates of heavy and light chain lineages of HIV-neutralizing antibodies (39). Furthermore, parameters describing population size, the speciation and extinction of species, and tree age can be further inferred, providing a set of parameters that lends itself easily to the comparison both within a single host and across different individuals.

### Toward systems phylogeny of antibody repertoires

The aforementioned metrics to quantify phylogenetic trees require just a single phylogenetic tree as input. The values arising from multiple trees can then be collectively analyzed to describe the selective pressure exerted upon the antibody repertoire as a whole. This traditional manner of studying the collection of antibody lineages, however, assumes a significant degree of independence between each phylogenetic tree. In an attempt to describe the population of antibody lineage trees, the UniFrac metric was applied to quantify the divergent evolution of immune systems arising during aging (35). The UniFrac metric was originally developed to measure the distance between microbial communities based on which branches are present in each sample, presenting a community-based statistic that can be easily adapted to Ig-Seq data (117). Another recent study aiming to characterize the dynamics of BCR evolution during HIV infection developed statistical models to describe clonal competition across multiple antibody lineages (118). Taken in concert, these studies represent important steps in the direction of statistics and analyses capable of describing the dynamic nature and evolution of antibody repertoire forests.

## Conclusion

Quantifying how antibody repertoires change over time represents an emerging field only possible due to the increased resolution of HTS and Ig-Seq. While the earliest phylogenetic metrics specifically tailored to antibody repertoire evolution were developed over a decade ago, more work remains necessary to comprehensively incorporate our experimental knowledge of antibodies into clonal lineage assignment, phylogenetic tree inference, and downstream analyses. Furthermore, benchmarking the aforementioned tools and strategies on both Ig-Seq data and multiple simulation frameworks can identify biases arising from the currently employed methodologies. The usage of lineage trees has immediate applications with medicinal relevance, such as vaccine design by targeting intermediate sequences or the discovery of therapeutic monoclonal antibodies. Furthermore, phylogenetics provides a unique opportunity to describe the clonal selection and competition underlying the pathogen-driven evolution of B cells. While phylogenetics has long held a role in the field of antibody research, the full potential of systems phylogenetics to delineate the complex co-evolving landscape between several independent lineages has not been realized. Other research fields such as machine learning, statistical entropy, and network analysis are becoming integral in antibody repertoire analysis, reinforcing the potential for phylogenetics to similarly take the stage to help delineate the complex picture of the B cell immunity.

## Author contributions

AY and SR conceived and designed the review. All authors wrote the review.

### Conflict of interest statement

The authors declare that the research was conducted in the absence of any commercial or financial relationships that could be construed as a potential conflict of interest.
